# Motor modules of human locomotion: influence of EMG averaging, concatenation, and number of step cycles

**DOI:** 10.3389/fnhum.2014.00335

**Published:** 2014-05-23

**Authors:** Anderson S. Oliveira, Leonardo Gizzi, Dario Farina, Uwe G. Kersting

**Affiliations:** ^1^Department of Health Science and Technology, Center for Sensory-Motor Interaction, Aalborg UniversityAalborg, Denmark; ^2^Pain Clinic Center for Anesthesiology, Emergency and Intensive Care Medicine, University Hospital GöttingenGöttingen, Germany; ^3^Department of Neurorehabilitation Engineering, Bernstein Focus Neurotechnology Göttingen, Bernstein Center for Computational Neuroscience, University Medical Center Göttingen, Georg-August UniversityGöttingen, Germany

**Keywords:** locomotion, variability, EMG, muscle synergies, motor modules, neural control

## Abstract

Locomotion can be investigated by factorization of electromyographic (EMG) signals, e.g., with non-negative matrix factorization (NMF). This approach is a convenient concise representation of muscle activities as distributed in motor modules, activated in specific gait phases. For applying NMF, the EMG signals are analyzed either as single trials, or as averaged EMG, or as concatenated EMG (data structure). The aim of this study is to investigate the influence of the data structure on the extracted motor modules. Twelve healthy men walked at their preferred speed on a treadmill while surface EMG signals were recorded for 60s from 10 lower limb muscles. Motor modules representing relative weightings of synergistic muscle activations were extracted by NMF from 40 step cycles separately (EMG_SNG_), from averaging 2, 3, 5, 10, 20, and 40 consecutive cycles (EMG_AVR_), and from the concatenation of the same sets of consecutive cycles (EMG_CNC_). Five motor modules were sufficient to reconstruct the original EMG datasets (reconstruction quality >90%), regardless of the type of data structure used. However, EMG_CNC_ was associated with a slightly reduced reconstruction quality with respect to EMG_AVR_. Most motor modules were similar when extracted from different data structures (similarity >0.85). However, the quality of the reconstructed 40-step EMG_CNC_ datasets when using the muscle weightings from EMG_AVR_ was low (reconstruction quality ~40%). On the other hand, the use of weightings from EMG_CNC_ for reconstructing this long period of locomotion provided higher quality, especially using 20 concatenated steps (reconstruction quality ~80%). Although EMG_SNG_ and EMG_AVR_ showed a higher reconstruction quality for short signal intervals, these data structures did not account for step-to-step variability. The results of this study provide practical guidelines on the methodological aspects of synergistic muscle activation extraction from EMG during locomotion.

## Introduction

Surface electromyography (EMG) represents indirectly the neural inputs from many sources (supraspinal, reflex activities, somatosensory information) to the muscles and has therefore been widely used to define neural strategies to perform motor tasks (Lacquaniti et al., 2012). An increasing number of investigations have been applying factorization analyses on multi-muscle surface EMG signals in order to extract basic motor patterns or modules (also called muscle synergies) that concisely represent the neural strategies for recruiting muscles during locomotor tasks (Ivanenko et al., 2005; Cappellini et al., 2006; Lacquaniti et al., 2012; Oliveira et al., 2013a). These investigations reported a low-dimensional model for representing the neural control of muscles during human locomotion, which is characterized by activation signals that define the instants of recruitment of specific motor modules related to biomechanical sub-tasks (Lacquaniti et al., 2012).

Human locomotion is a largely automatized motor behavior, therefore the step-to-step variability of the main neural inputs to the muscles is limited. Studies applying factorization analysis focusing on human locomotion usually report a low-dimensional set of four to six motor modules to represent neural inputs to the muscles (Ivanenko et al., 2004; Lacquaniti et al., 2012). Differences in the number of motor modules needed for an accurate description (i.e., dimensionality) may be related to a variable number of muscles included in the EMG dataset and different low-pass filtering among studies (Hug et al., 2012; Steele et al., 2013). In addition, there is a wide range of number of step cycles used for extracting representative motor modules; for example, some studies used 4–12 cycles (Monaco et al., 2010), others 10–25 cycles (Merkle et al., 1998; Ivanenko et al., 2004, 2005; Gizzi et al., 2011; Oliveira et al., 2013a; Sartori et al., 2013b), and in some cases up to 30 cycles (Clark et al., 2010). The number of step cycles used for the estimation of the synergistic activation is relevant for applications to biofeedback and rehabilitation technologies, when the extracted motor modules are used either for feedback purposes or as a basis for controlling the interaction with robotic devices. For example, because the motor modules provides a concise representation of relative muscle activations, their online estimation can be used by a training supervisor to focus the attention of the patient on those muscles that are abnormally activated during a certain phase of the step cycle.

Previous investigations extracting motor modules from single trials reported reconstruction quality over 90% (Ivanenko et al., 2004, 2005), whereas analyses in which consecutive step cycles were concatenated and analyzed together resulted in a lower reconstruction quality (Gizzi et al., 2011; Oliveira et al., 2012, 2013a,b). Reduced reconstruction quality in concatenated analyses may be an effect of natural step-to-step variability contained in surface EMG signals, which may be crucial for specific kinematic adjustments during locomotion. Recently, de Rugy et al. (2013) have raised concerns about the use of factorization analysis because they noticed that even small reconstruction errors in muscle activity could correspond to relatively important changes in force production. Therefore, although EMG factorization analysis is a promising tool for locomotion rehabilitation and robotic control (Gizzi et al., 2012; Moreno et al., 2013; Sartori et al., 2013a), there are many aspects that still remain unclear for an optimal and consistent application of such methodology.

In this study we explored the differences in the extracted motor modules when varying the EMG data structure by comparing the factorization results when using single step EMG (EMG_SNG_), averaged EMG (EMG_AVR_), and concatenated EMG (EMG_CNC_). Applications in neurotechnologies for rehabilitation, e.g., biofeedback, would benefit from the analysis on the shortest time interval (single cycles) that would allow adaptive/reactive responses. However, single cycle factorization would present fast variations on a cycle basis. These variations may be relevant in some applications, e.g., in patients with high intrinsic step-to-step variability, but not in others. The hypothesis of the study was that the use of different data structures (single trials, averaging or concatenating EMG signals) to identify motor modules influences the extracted dimensionality and/or the modules. The results obtained are of practical relevance when using EMG factorization for the study of human locomotion.

## Methods

### Subjects

Twelve healthy men (age: 28 ± 4 years; body mass: 80.8 ± 8 kg; body height: 178 ± 4 cm) volunteered for the experiment. One subject was left-dominant and all others were right-dominant. Exclusion criteria included history of knee or ankle ligament injury, current lower-extremity injury, recent (within 6 months) low back injury, or vestibular dysfunction. All subjects provided written informed consent before participation and the procedures were approved by the ethical committee of Northern Jutland (N-20130015).

### Experimental setup

**Figure 1:**
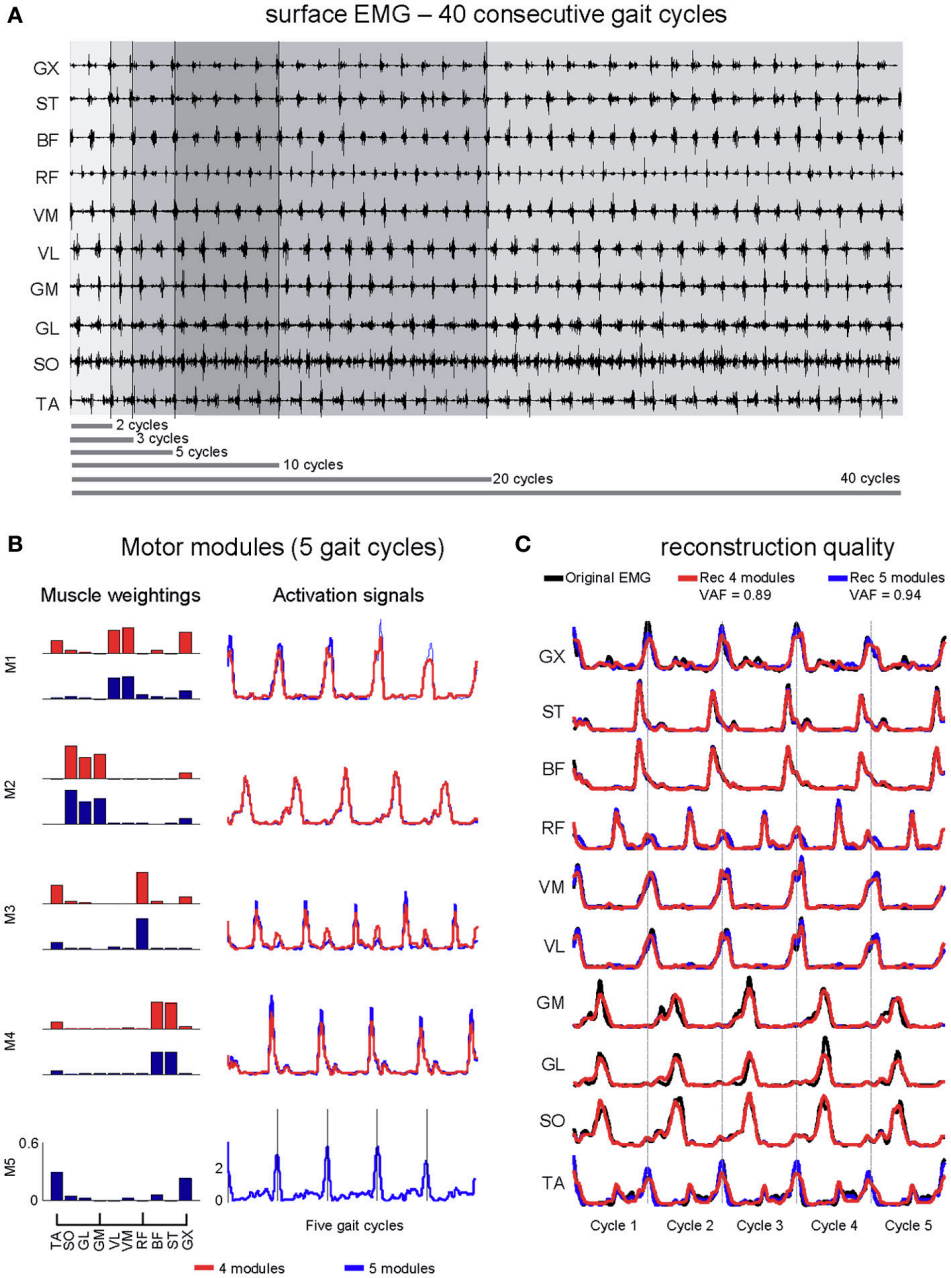
**Illustrative sample of surface EMG signals (A) from 40 consecutive walking step cycles. (B)** Non-negative matrix factorization provides similar muscle weightings by using 4 and 5 motor modules (similarity >0.9 for the first four motor modules), however differences in the activation signals can be qualitatively observed, especially for M1 and M3. The reconstruction of the original EMG dataset is shown in **(C)**. The original EMG (*black lines*) from some muscles (i.e., VL, VM, BF, ST) is well reconstructed by using both 4 modules (Rec 4 modules, *red lines*) or five modules (Rec 5 modules, *blue lines*). On the other hand, muscles such as TA, RF, and GX exhibited better reconstruction only by using 5 modules.

In a single session subjects were initially asked to perform familiarization to the treadmill (Woodway Pro, Foster Court Waukesha, USA) by walking for 5 min. Subsequently, preferred walking speed was determined following previous literature (Choi and Bastian, 2007) and after a 2-min rest period, subjects walked at the selected speed for 5 min during which surface EMG and walking cadence were recorded from the last 60s (see Figure 1A for illustration).

### Data collection

EMG signals were recorded in bipolar derivations with pairs of Ag/AgCl electrodes (Ambu Neuroline 720 01-K/12; Ambu, Ballerup, Denmark) with 22 mm of center-to-center spacing. Prior to electrode placement the skin was shaved and lightly abraded. A reference electrode was placed on the right tibia. The EMG signals were recorded from a portable EMG amplifier (Biovision EMG-Amp, Germany) stored in a backpack together with a mini-computer. The EMG signals were sampled at 2000 Hz (12 bits per sample), band-pass filtered (second-order, zero lag Butterworth, bandwidth 10–500 Hz). The EMG signals were recorded from the following muscles of the right side (dominant side for 11 out of 12 subjects) according to Barbero et al. (2011): tibialis anterior (TA), soleus (SO), gastrocnemius lateralis (GL), gastrocnemius medialis (GM), vastus lateralis (VL), vastus medialis (VM), rectus femoris (RF), biceps femoris (BF), semitendinosus (ST), and gluteus maximus (GX). A uniaxial accelerometer was placed on the right tibia, which measured the vertical acceleration synchronized to the EMG measurements.

### Data analysis

#### Accelerometry

Data from tibia vertical acceleration were low-pass filtered (60 Hz) and step cycles were determined following previously reported methods (Kersting, 2011). Individual step cycles were time-normalized to 200 data points for one step cycle.

#### Surface EMG

The segmentation for EMG factorization was defined from the accelerometer data, from which step cycles were determined. After segmentation, the surface EMG signals from the 10 muscles were band-pass filtered (20–500 Hz), full-wave rectified, low-pass filtered (10 Hz) and time-normalized in order to obtain 200 data points for one step cycle.

#### Motor modules extraction

For each subject, non-negative matrix factorization (NMF, Lee and Seung, 2001) was applied in order to process the EMG_SNG_ extraction and identify motor modules and activation signals from the 40 consecutive step cycles separately. Subsequently, the vectors representing muscle weightings and activation signals were averaged for each subject which could be compared to motor modules from the other two processing methods. In addition, NMF was applied in EMG datasets containing two, three, five, 10, 20, and 40 consecutive step cycles in two processing modalities. The first (EMG_AVR_) consisted of averaging the different number of step cycles for subsequent extraction of motor modules. The second method (EMG_CNC_) consisted of the concatenation of a given amount of step cycles for subsequent extraction of motor modules. In this case, all variability from sequential step cycles is accounted for during NMF analysis, which may reduce reconstruction quality for longer datasets including a greater number of consecutive cycles (see Figures 1B,C for illustration). For all three EMG processing methods, individual surface EMG channels were normalized by the peak activation, so that all channels were ranging from 0 to 1 in amplitude.

#### Motor module model

The EMG signals *X(k)* recorded from *M* muscles were factorized as

(1) $$X(k)\approx X_{r}(k)=S\cdot P(k)$$

where *X*_*r*_(*k*) is the muscle activity vector reconstructed by the factorization, *S* is a scalar matrix (synergy matrix or motor module matrix), and *P(k)* are the activation signals, of dimension *N* < *M*. In Equation (1), the EMG *X(k)* are obtained by linear transformation of the activation signals *P(k)* with gain factors *s*_*mn*_ (the entries of the synergy matrix, Lee and Seung, 2001).

#### Dimensionality

After extracting the motor modules, the estimated muscular activation pattern was compared with the experimental pattern by means of the variability accounted for (VAF) value, defined as the variation that can be explained by the model: VAF = 1− SSE/SST, where SSE (sum of squared errors) is the unexplained variation and SST (total sum of squares) is the pooled variation of the data. The reconstruction quality was analyzed by plotting the VAF as a function of the number of modules, and the minimum acceptable number of modules was identified as the point in which this curve pronouncedly changes its slope (d'Avella et al., 2003; Muceli et al., 2010), and additionally, the number of modules must also successfully reconstruct at least 90% of the original EMG content. In addition, we reconstructed EMG signals from the three processing methods and number of steps in two different ways: (a) the combination of extracted muscle weightings with activation signals obtained from randomly generated matrix (i.e., activation signals free to vary) and (b) the combination of extracted activation signals with muscle weightings obtained from randomly generated matrix (i.e., muscle weightings free to vary). The latter analysis provided the quality of EMG reconstruction (i.e., VAF) that can be achieved by using random variability, which was hypothesized to be lower than the VAF obtained by reconstructing EMG signals with the factorization obtained by NMF. The muscle weightings and activation signals free to vary were obtained by iterating 1000 times the NMF update rules (Lee and Seung, 1999), only for muscle weightings or activation signals, respectively.

#### Similarities

The muscle weightings and activation signals from two sets were compared by computing the similarity between the best matched pairs, as described in d'Avella et al. (2003). Similarities were then calculated by computing the scalar product between pairs of vectors (motor modules or activation signals), normalized by the product of the norms of each column (d'Avella et al., 2003; Muceli et al., 2010), which prioritizes the comparison between the shapes of vectors rather than amplitude. Similarity can vary from 0 (no curve shape matching) to 1 (perfect curve shape matching) and previous investigations have used values above 0.8 to define if a pair of vectors is similar (Gizzi et al., 2011; Oliveira et al., 2013b). Intra-subject similarity analyses were conducted for individual motor modules and individual activation signals between the different numbers of step cycles for each given EMG processing method. In addition, intra-subject similarities between methods were calculated for each sequence of step cycles.

#### EMG reconstructed from different muscle weightings

Additionally to similarity analysis, we fixed the muscle weightings extracted from 2, 3, 5, 10, and 20 steps of the first half of the recording, and used such weightings for reconstructing another sequence of 2, 3, 5, 10, and 20 concatenated steps from the second half, as well as the whole sequence of 40 cycles. This analysis reflected the situation in which the motor modules are computed from only an initial portion of the recording and then used to explain the remaining part of the recording. This procedure was conducted by using muscle weightings from EMG_CNC_ and from EMG_AVR_. For instance, we reconstructed the concatenated EMG from 40 step cycles by using its original activation signals combined to the muscle weightings from shorter concatenation periods (2, 3, 5, 10, and 20 cycles). By doing so we aimed at directly testing the reconstruction performance of motor modules extracted from different concatenation lengths as a measure of their representativeness for a longer signal interval.

#### Statistical analysis

The degrees of similarity between individual motor modules and between individual activation signals were compared by a One-Way ANOVA. The significance level was set to *p* < 0.05. A Two-Way ANOVA was used in order to verify the effects of EMG processing method (EMG_AVR_ vs. EMG_CNC_) and number of step cycles (two, three, five, 10, 20, and 40) on the reconstruction quality (VAF). In addition, Student *t*-tests were used to investigate differences between intra-subject similarities among the EMG processing methods. All statistical procedures were conducted using SPSS 18.0 (SPSS, Inc., Chicago, IL, USA).

## Results

### Dimensionality

**Figure 2:**
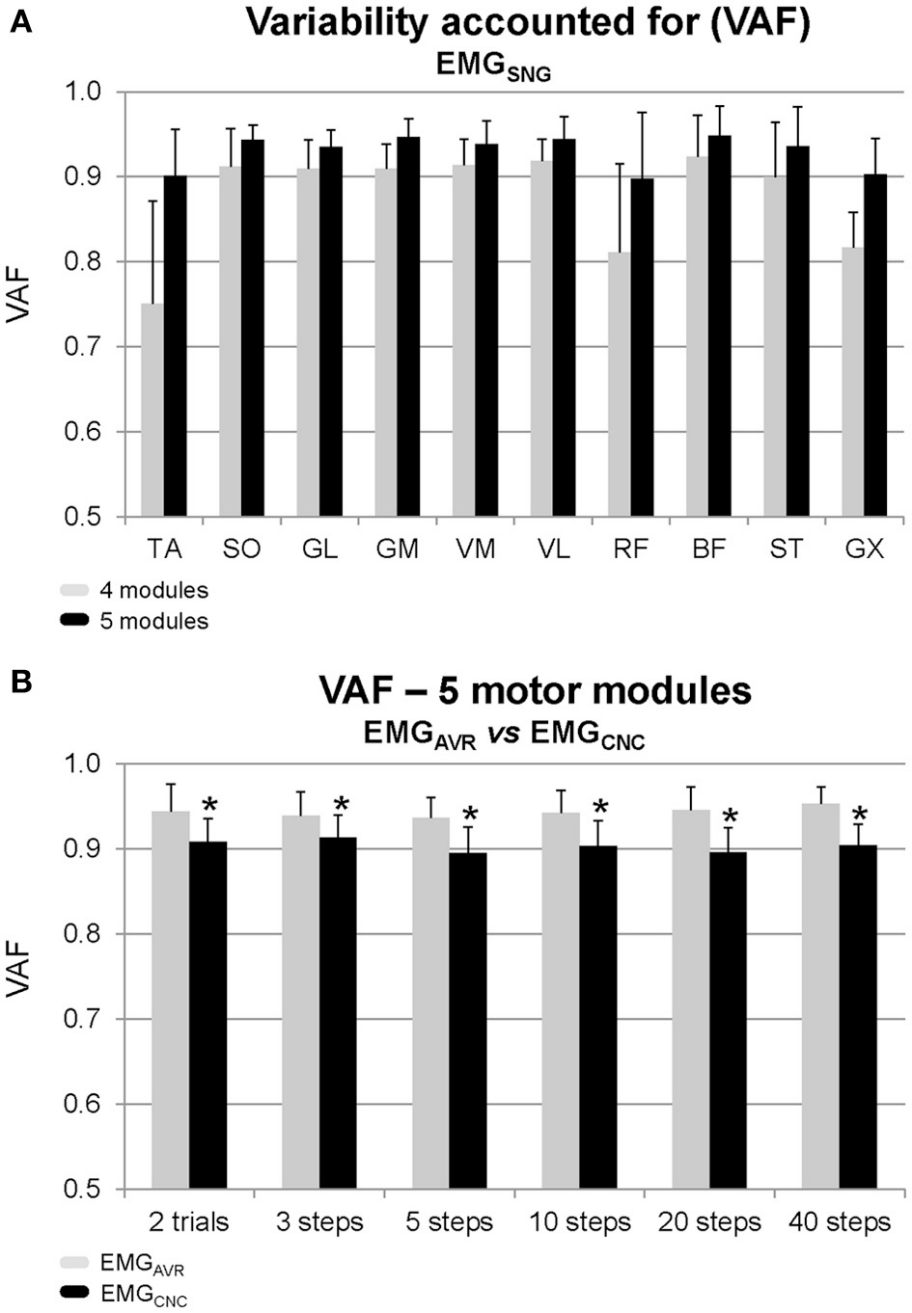
**Mean ± *SD* of the variation accounted for (VAF) from the factorization analysis of individual step cycles (A) by considering to reconstruct the original EMG dataset using four motor modules (*gray bars*) and five motor modules (*black bars*) from single step cycles (EMG_SNG_). (B)** The VAF from the reconstruction of original EMG datasets by using five motor modules from averaged EMG (EMG_AVR_, *gray bars*) and from concatenated (EMG_CNC_, *black bars*) in different amounts of step cycles. ^*^Denotes significant difference in relation to EMG_AVR_ (*p* < 0.05).

The analysis of dimensionality from EMG_SNG_ revealed that four motor modules were required to reconstruct unilateral muscular activations with an overall reconstruction quality of 88% (VAF = 88 ± 3%, average across all muscles, Figure 2A). For three out of 12 subjects the VAF was higher than 90% for all muscles by reconstructing the EMG from four motor modules. However, for most of subjects, muscles such as TA, RF, and GX showed poorer reconstruction quality than the average (~80%). By using five motor modules the overall reconstruction quality increased to 93 ± 2%, and all muscles could reach an average reconstruction quality >90% (see Figures 1B,C for illustration).

The calculated VAF by combining randomly generated muscle weightings and the extracted activation signals to reconstruct the original EMG datasets was 38 ± 8, 36 ± 11, and 32 ± 9% for EMG_SNG_, EMG_AVR_, and EMG_CNC_ respectively. Similarly, the results from calculating the VAF by combining randomly generated activation signals and the extracted muscle weightings to reconstruct the original EMG datasets was 39 ± 10%, 33 ± 12%, and 37 ± 10% for EMG_SNG_, EMG_AVR_, and EMG_CNC_ respectively. Both simulations showed a very poor reconstruction quality in comparison to the extracted motor modules, which suggests that the extracted motor modules provide meaningful information that random variability cannot reproduce.

### Averaging vs. concatenating EMG signals

By fixing the number of modules to five we compared the results from EMG_AVR_ and EMG_CNC_. The Two-Way ANOVA revealed no EMG processing vs. number of step cycles interaction, however there was a significant effect of the EMG processing method (*p* < 0.001, *F* = 90.5). The reconstruction accuracy was approximately 94% (VAF = 0.94 ± 0.02, Figure 2B) for motor modules from EMG_AVR_, and slightly lower (~90%) when using EMG_CNC_ (VAF = 0.90 ± 0.03). In both cases, the number of step cycles used for the calculation did not significantly influence the estimates.

### Motor modules from treadmill walking

**Figure 3:**
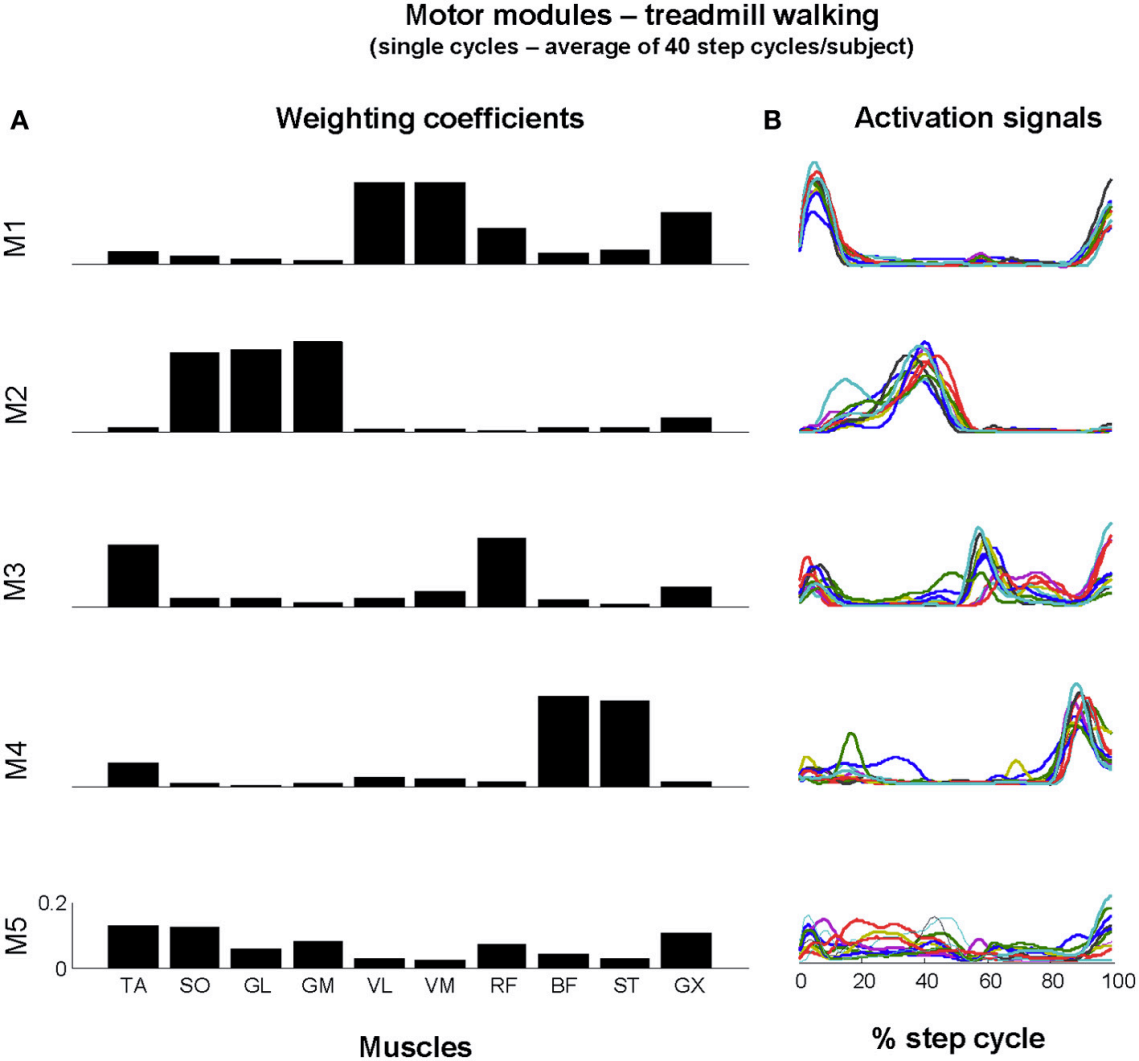
**Motor modules extracted from EMG_AVR_ of all subjects concatenated**. Muscle weightings were averaged for illustration **(A)** and each activation signal for single subjects (each subject represented by a color) is shown in **(B)**. The first four activation signals exhibited well-defined bursts of activity at specific timing, however M5 did not presented clear pattern of activation throughout the step cycle, as well as between subjects.

Four out of the five motor modules could be assigned to biomechanical subtasks of walking (Figure 3). Module 1 (M1) consists of the activation of knee extensors and GX (see muscle weightings in Figure 3A) at the beginning of the stance period (see activation signals in Figure 3B). Module 2 (M2) relates to forward propulsion, in which the plantarflexors are predominantly recruited. Module 3 (M3) relates to the leg swing, in which TA and RF are recruited throughout the swing phase, and Module 4 (M4) is related to the recruitment of the hamstring muscles (ST, BF) prior to the subsequent initial contact. The fifth module (M5) involves the recruitment of ankle joint muscles as well as RF and GX, with no clear burst-like activity throughout the step cycle. The recruitment of this motor module is predominant at initial contact, transition from stance to swing phase and prior to subsequent initial contact.

### Intra-subject similarities

High similarities considering all ranges of step cycles (>0.8) were found for individual muscle weightings and individual activation signals of all motor modules except M5, regardless the used EMG processing method for motor modules extraction (Table 1). However, EMG_SNG_ exhibited reduced intra-subject similarity for individual muscle weightings in comparison to EMG_AVR_ and EMG_CNC_ for most of the modules (ANOVA One-Way, *p* < 0.05). In addition, EMG_SNG_ also exhibited reduced intra-subject similarity for individual activation signals in comparison to EMG_AVR_ for all motor modules (*t*-Student test, *p* < 0.05).

**Table 1:** **Mean ± *SD* intra-subject similarities for each motor module (M1–M5) extracted by using surface EMG from single step cycles (EMG_SNG_), averaged EMG (EMG_AVR_), and concatenated EMG (EMG_CNC_)**.

	**M1**	**M2**	**M3**	**M4**	**M5**
**MUSCLE WEIGHTINGS**					
EMG_SNG_	0.89±0.10^*^	0.89±0.11^*^	0.86±0.13	0.85±0.03^*^	0.58±0.14^*^
EMG_AVR_	0.97±0.02	0.97±0.02	0.92±0.12	0.98±0.01	0.78±0.18
EMG_CNC_	0.94±0.06	0.96±0.02	0.86±0.20	0.97±0.02	0.78±0.19
**ACTIVATION SIGNALS**					
EMG_SNG_	0.92±0.08^*^	0.93±0.03^*^	0.82±0.10^*^	0.92±0.06^*^	0.60±0.10^*^
EMG_AVR_	0.99±0.01	0.97±0.02	0.91±0.12	0.96±0.05	0.79±0.17

For each subject similarity was computed across all ranges of step cycles, therefore it was not possible to compute similarities for the concatenated activation signals.

* Denotes significant difference in relation to the other EMG processing methods.

### Similarity among EMG processing methods

**Figure 4:**
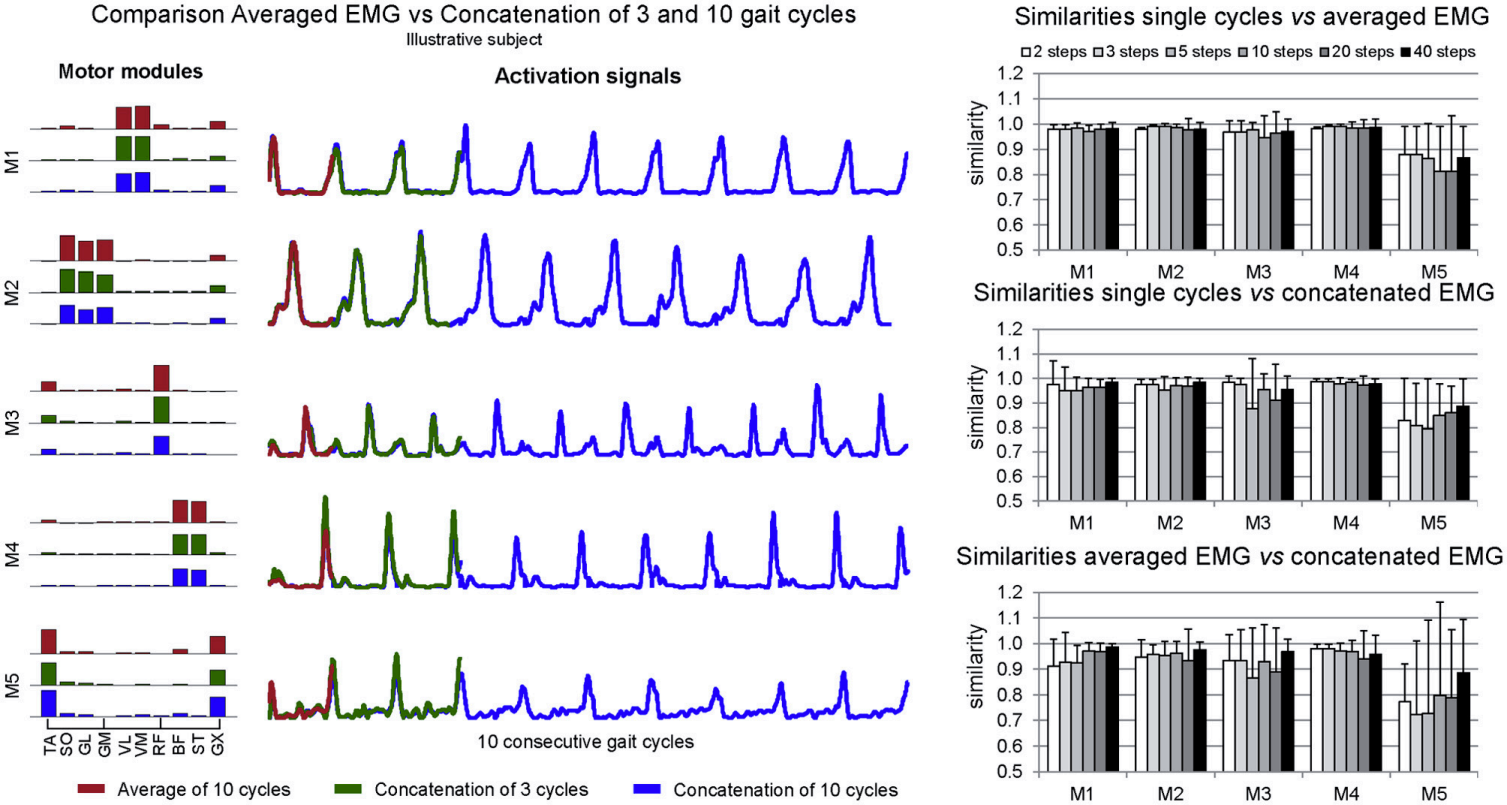
**Illustrative comparison of averaged EMG and concatenated EMG from a single subject (*left*)**. On the right, mean ± *SD* similarities between muscle weightings extracted from single trials vs. averaged EMG (*top*), from single trials vs. concatenated EMG (*middle*) and from averaged EMG vs. single trials (*bottom*), for each analyzed number of step cycles (from three to 40 step cycles).

Intra-subject similarity among methods (Figure 4) was high between EMG_SNG_ vs. EMG_AVR_ (similarity = 0.95 ± 0.09 considering all ranges of cycles numbers and the five motor modules), as well as between EMG_SNG_ vs. EMG_CNC_ (similarity = 0.94 ± 0.10). A 1-way ANOVA test for each motor module did not reveal any statistical differences (*p* > 0.05). Similarity between EMG_AVR_ vs. EMG_CNC_ was slightly reduced (0.92 ± 0.16), especially for M5 (0.80 ± 0.15). For the motor module related to leg swing (M3) similarity between EMG_SNG_ vs. EMG_AVR_ (0.96 ± 0.01) was slightly higher than the similarity between EMG_SNG_ vs. EMG_CNC_ (0.93 ± 0.01) and EMG_AVR_ vs. EMG_CNC_ (0.90 ± 0.01).

### Reconstructed EMG from different concatenations of step cycles

**Figure 5:**
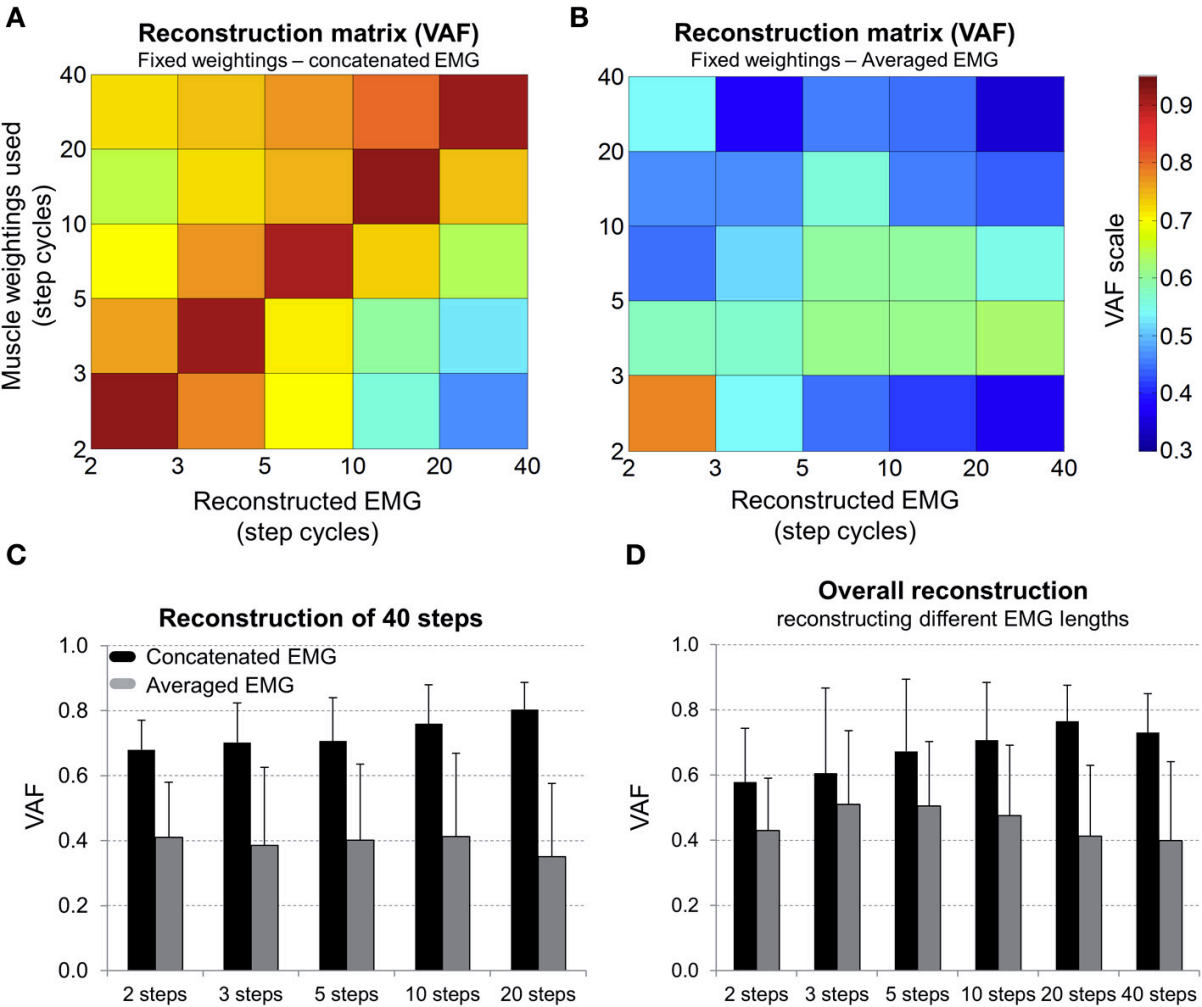
**(A)** Colormap representing the variability accounted for (VAF) the reconstruction of surface EMG weightings by combining fixed muscle weightings from a given number of step cycles to the activation signals of a given number of step cycles. **(B)** Mean (SD) VAF of reconstructing EMG from 40 concatenated step cycles by using muscle weightings from the concatenation of 2–20 steps. **(C)** Mean (SD) VAF of reconstructing an entire 40 step cycles EMG dataset by using muscle weightings from the concatenation (*black bars*) and from the average of 2–20 continuous step cycles (*gray bars*). **(D)** Mean (SD) VAF displaying overall reconstruction quality achieved by using muscle weightings from the concatenation (*black bars*) and average (*gray bars*) of specific number of step cycles (from 2 to 40) to reconstruct all other step ranges.

The reconstruction of original EMG using muscle weightings from EMG_CNC_ revealed that the lower the number of concatenated step cycles, the lower is the quality of reconstruction for longer concatenation periods (Figure 5A). On the other hand, the use of weightings from EMG_AVR_ did not provide a similar reconstruction quality (Figure 5B). In a more detailed analysis concerning the reconstruction of 40 steps (Figure 5C), it was observed that the use of muscle weightings from EMG_CNC_ provided higher reconstruction quality in comparison to EMG_AVR._ Moreover, the highest reconstruction quality was achieved by using the concatenation of 20 steps (VAF = 0.8 ± 0.04, Figure 5C). In addition, the overall quality of reconstruction by using a given number of step cycles to reconstruct the EMG datasets of the different number of steps is shown in Figure 5D. It was also observed that EMG_CNC_ provided higher reconstruction quality in comparison to EMG_AVR_, and especially for EMG_CNC_ the shorter the concatenation period, the poorer is the quality of reconstruction.

## Discussion

We studied the influence of the data structure (e.g., number of step cycles) and their processing (averaging/concatenation) on the EMG factorization analysis during locomotion. The results indicated that the number of step cycles and their pre-processing did not impact the estimated dimensionality and had a relatively small effect on the extracted motor modules, as intra-subject similarities demonstrated that these motor modules were predominantly similar regardless of the number of analyzed step cycles. However, further analyses applying muscle weightings from EMG_AVR_ and shorter EMG_CNC_ intervals to reconstruct longer locomotion intervals (e.g., 40 step cycles) demonstrated poor reconstruction quality, while optimal reconstructions were found by using at least 20 steps.

As expected, the extraction of motor modules from individual step cycles revealed a certain degree of step-to-step variability in the results. Because of this variability, when muscle weightings extracted from EMG_AVR_ or from EMG_CNC_ with small number of step cycles were fixed for reconstructing the original EMG data from different concatenation periods, the reconstruction quality was generally poor. These results suggest that, although different EMG processing methods can reveal predominantly similar vectors for weightings and timing properties, the details of muscle activities and their variability are better extracted by concatenating at least 20 step cycles. This number of step cycles, when concatenated, allowed to capture most of the step-by-step variability generated during longer periods of locomotion.

The reconstruction of original EMG datasets by using four motor modules was not high for TA, RF, and GX in most of the tested subjects. However, the four extracted modules were still consistent when five modules were extracted, and were comparable to those reported in previous literature (Ivanenko et al., 2004; Clark et al., 2010; Oliveira et al., 2012). In our study, a fifth motor module that was not directly associated to a biomechanical subtask, was required to complement the activation of ankle joint muscles, and of the RF and GX muscles at the transition instances of the step cycle (swing-to-stance and stance-to-swing). This type of motor modules with relatively small biomechanical relevance has been previously reported by Monaco et al. (2010) who defined it as systematic information with robust inter-subject muscle groups, especially at high cadences. Ivanenko et al. (2004, 2005) also described less relevant motor patterns that could be dropped from the analysis because of their lack of significance. In the present study, the fifth motor module could only capture a marginal portion of the EMG variance and may not be necessary to understand the main features of the global EMG data. However, the consistently lower reconstruction quality for the same muscles when using four modules may indicate that additional temporal adjustments in muscle recruitment might be needed in order to produce optimal limb kinematics. The exact source of such activity can only be speculated, involving sensorial/afferent inputs to the muscles (Rossignol et al., 2006) and/or direct modulation from cortical neurons (Petersen et al., 2012). Gwin et al. (2011) have shown increased spectral power in the alpha and beta bands of cortical activity during step transitions, the predominant periods in which the described fifth module was recruited. Therefore, these additional components should not be disregarded while extracting motor modules if the purpose of the experiment requires high-quality EMG reconstruction.

The high similarity observed across EMG processing methods, including different numbers of step cycles, may initially suggest that single steps can be representative of all variability contained in longer locomotion periods. Therefore, we also analyzed the intra-subject variability that motor modules exhibited with the different EMG processing methods. This analysis showed a reduced similarity among the extracted motor modules from EMG_SNG_ in comparison to the other methods, suggesting that individual motor modules from EMG_SNG_ do not contain sufficient EMG variability for representing the EMG step pattern. When using the EMG_AVR_ or EMG_CNC_ datasets, the variability of the entire recording was included, either by averaging or by factorizing the whole signal interval, which explains the higher intra-subject similarity for these EMG processing methods. Although these results from longer ambulation periods were superior than those extracted from single trials, we also used a cross-validation procedure for verifying if the weightings could be shared between concatenation periods while generating successful reconstruction (Oliveira et al., 2013b). The use of muscle weightings from the concatenation of less than 10 step cycles reconstructed the original EMG from 40 step cycles by less than 70% whereas, when using 20 cycles, the VAF raised to 80% on average. This result suggests that a rather long locomotion period is preferable to optimally represent the modular organization of human locomotion and its variability over time. Interestingly, the use of weightings from EMG_AVR_ did not reach the same reconstruction quality as those from EMG_CNC_, even though most of the weightings from these two conditions presented similarities above 90%. This observation may indicate the limitation of this similarity measure. Therefore, the comparison of results from factorization analysis of different tasks may not be exclusively based on similarity indexes, and the use of a cross-validation method such as fixing the muscle weightings in combination with timing properties of the signal to be reconstructed may be more valuable.

In the present investigation we recorded 10 lower limb muscles directly involved in locomotor mechanics. Previous investigations have recorded the EMG activity from up to 32 muscles and found five principal components that modulate muscle recruitment (Ivanenko et al., 2005), while other studies containing fewer muscles reported four motor modules (McGowan et al., 2010; Monaco et al., 2010; Gizzi et al., 2011). Our results are therefore in agreement with these previous reports and we speculate that the addition of other muscles such as hip extensors, adductors, and abductors may lead to an increased dimensionality. However the outcome of the methodological comparisons may be preserved if the results are extracted from locomotion at constant speed. The use of treadmill walking in this investigation provided an ideal model of locomotion in a controlled environment and at a fixed speed. The lack of kinematic measurements is a limitation of this investigation, however there is an extensive body of literature describing walking kinematics and its variability, and its relationship to EMG variability (Winter and Yack, 1987; Ivanenko et al., 2002; Kang and Dingwell, 2006). Indeed, despite the considerable EMG variability during locomotion, lower limb kinematics appear less variable (Winter and Yack, 1987; Ivanenko et al., 2002; Kang and Dingwell, 2006) due to inertial and damping properties of body segments that smoothen individual muscle force fluctuations (Kang and Dingwell, 2006, 2009). This observation supports the conclusion that muscle recruitment can be essentially modulated to control the overall limb kinematics (Ivanenko et al., 2002). Another limitation of this study is the subject sample of only healthy subjects. The results obtained may not be entirely applicable for clinical cases in which there is more variability in kinematics and muscle recruitment (Clark et al., 2010; Gizzi et al., 2011).

In summary, the present investigation showed that the dimensionality of motor modules was not influenced by the number of step cycles used for EMG factorization. We also noted that the dimensionality must be accurately defined such that the reconstruction of all the involved muscles reaches acceptable levels. In this experiment, four motor modules could account for most of the EMG variability and could be assigned to biomechanical subtasks, but for optimal muscle reconstruction a fifth motor module was required. In addition, although muscle weightings from the factorization of different numbers of step cycles and processing methods are predominantly similar, the use of muscle weightings from the factorization of a sufficient number of concatenated step cycles can better represent locomotion over longer periods.

## Author contributions

Anderson S. Oliveira, Leonardo Gizzi, Uwe G. Kersting, and Dario Farina designed the experiment. Anderson S. Oliveira and Uwe G. Kersting performed the experiments. Anderson S. Oliveira, Leonardo Gizzi, and Dario Farina analyzed and interpreted the data. Anderson S. Oliveira, Leonardo Gizzi, Uwe G. Kersting, and Dario Farina drafted the manuscript and all authors approved the final version. Experiments were performed at Aalborg University.

### Conflict of interest statement

The authors declare that the research was conducted in the absence of any commercial or financial relationships that could be construed as a potential conflict of interest.
